# Synergistic Toxicity of Pollutant and Ultraviolet Exposure from a Mitochondrial Perspective

**DOI:** 10.3390/ijms25179146

**Published:** 2024-08-23

**Authors:** Eloïse Larnac, Sébastien Méthot, Frédéric Pelchat, Marc-Antoine Millette, Alicia Montoni, Christian Salesse, Valérie Haydont, Laurent Marrot, Patrick J. Rochette

**Affiliations:** 1 Centre de Recherche du CHU de Québec, Université Laval, Axe Médecine Régénératrice, Hôpital du Saint-Sacrement, Québec, QC G1S 4L8, Canada; eloise.larnac.1@ulaval.ca (E.L.); sebastien.methot.1@ulaval.ca (S.M.); frederic.pelchat.2@ulaval.ca (F.P.); marc-antoine.millette.1@ulaval.ca (M.-A.M.); christian.salesse@fmed.ulaval.ca (C.S.); 2 Centre de Recherche en Organogénèse Expérimentale, Université Laval/LOEX, Québec, QC G1V 0A6, Canada; alicia.montoni@crchudequebec.ulaval.ca; 3 Faculté de Médecine, Département d’Ophtalmologie, Université Laval, Québec, QC G1V 0A6, Canada; 4 Advanced Research, L’OREAL Research & Innovation, 93600 Aulnay-Sous-Bois, France; valerie.haydont@loreal.com (V.H.); laurent.marrot@rd.loreal.com (L.M.)

**Keywords:** ultraviolet, pollution, photo-pollution, mitochondria, benzo[a]pyrene

## Abstract

Ultraviolet (UV) exposure and atmospheric pollution are both independently implicated in skin diseases such as cancer and premature aging. UVA wavelengths, which penetrate in the deep layers of the skin dermis, exert their toxicity mainly through chromophore photosensitization reactions. Benzo[a]pyrene (BaP), the most abundant polycyclic aromatic hydrocarbon originating from the incomplete combustion of organic matter, could act as a chromophore and absorb UVA. We and other groups have previously shown that BaP and UVA synergize their toxicity in skin cells, which leads to important oxidation. Even if mitochondria alterations have been related to premature skin aging and other skin disorders, no studies have focused on the synergy between UV exposure and pollution on mitochondria. Our study aims to investigate the combined effect of UVA and BaP specifically on mitochondria in order to assess the effect on mitochondrial membranes and the consequences on mitochondrial activity. We show that BaP has a strong affinity for mitochondria and that this affinity leads to an important induction of lipid peroxidation and membrane disruption when exposed to UVA. Co-exposure to UVA and BaP synergizes their toxicity to negatively impact mitochondrial membrane potential, mitochondrial metabolism and the mitochondrial network. Altogether, our results highlight the implication of mitochondria in the synergistic toxicity of pollution and UV exposure and the potential of this toxicity on skin integrity.

## 1. Introduction

Mitochondria are known for their role in providing adenosine triphosphate (ATP) to cells, but they are also implicated in many other processes, including apoptosis, the production of reactive oxygen species (ROS) and calcium homeostasis [1,2,3]. Different environmental factors are known to alter mitochondria, leading to an abnormal production of ROS. When stress is extensive and/or persistent, it can promote metabolic or neurodegenerative diseases [4]. It has also been shown that mitochondrial alterations can lead to premature skin aging [5,6,7,8].

Among the environmental factors implicated in dermal aging, chronic exposure to UVA is considered the most important. Numerous studies have shown that chronic UVA exposure alters mitochondrial DNA (mtDNA) and creates various mitochondrial mutations [5,9,10]. There is a hypothetical vicious cycle in which the accumulation of mtDNA mutations leads to mitochondrial dysfunctions and a consequent increase in oxidative stress. This oxidative stress then affects mitochondrial homeostasis and causes more mtDNA mutations, leading to increased ROS production, feeding the vicious circle [11]. UVA has also been shown to directly cause the loss of mitochondrial membrane potential [12], a decrease in ATP production and the alteration of mitochondrial respiration [13,14]. 

Pollution is increasingly seen as an environmental factor that affects mitochondria. Indeed, different studies have shown the effect of several pollutants on mitochondria [15,16,17]. These stressors lead to increased ROS content, disrupted mtDNA copy number and decreased membrane potential and ATP production and can induce mitochondrial fission. Among the studied pollutants, polycyclic aromatic hydrocarbons (PAHs) are abundant pollutants resulting from the incomplete combustion of organic matter. PAHs are ubiquitous, and we are continuously exposed to them through the inhalation of cigarette smoke, diesel exhaust, coal power plant emissions, wood combustion and forest fires and the ingestion of grilled food [18,19]. PAHs are lipophilic compounds with a condensate benzene ring structure. Their small size facilitates their translocation from pulmonary alveoli to capillaries after inhalation [18,20,21]. They are then transported into the whole body through blood circulation where they are found in the nanomolar range [22]. Among all PAHs, benzo[a]pyrene (BaP) is one of the most studied and has been recognized as carcinogenic to humans by the International Agency for Research on Cancer (IARC) [23]. BaP has been linked to different pathologies [24,25] including cancer [23] but also skin aging [19,22]. Different studies highlighted that BaP can impact mitochondria, leading to increased mitochondrial ROS production and to decreased mitochondrial membrane potential, ATP production and mtDNA copy number [24,26,27,28]. 

We and other groups have previously shown that BaP has the ability to act as a chromophore, absorbing UVA wavelengths [22,29,30]. Several studies have been carried out to better understand the potential toxic synergy between PAHs and UV rays on the skin [31,32,33,34,35]. However, very few studies investigated the synergistic effect of pollution and light on mitochondria. Das et al. looked at the combined effect of UVB and BaP in the context of skin cancers, showing an increased ROS induction and an increase in mitochondrial proteins implicated in apoptosis [36]. The team of Mokrzyński et al. [37] studied the synergy between PM2.5 and UVA on HaCaT cells and showed a loss of mitochondrial membrane potential and a change in gene expression related to mitochondrial-associated apoptosis. Soeur et al. [22] investigated the effect of two PAHs, BaP and indenopyrene (IcdP), on a UVA-irradiated reconstructed epidermis. They showed an increase in cellular oxidative stress and mitochondrial membrane depolarization and a resulting decreased ATP production. Thus, the mitochondrial implication in the toxic co-exposure to pollution and light has not been extensively studied. In this context, this study aims to investigate the combined effect of UVA and BaP specifically on mitochondria in order to assess the effect on mitochondrial membranes and the consequences on mitochondrial activity. 

## 2. Results 

### 2.1. Affinity of BaP for Mitochondria

**Figure 1:**
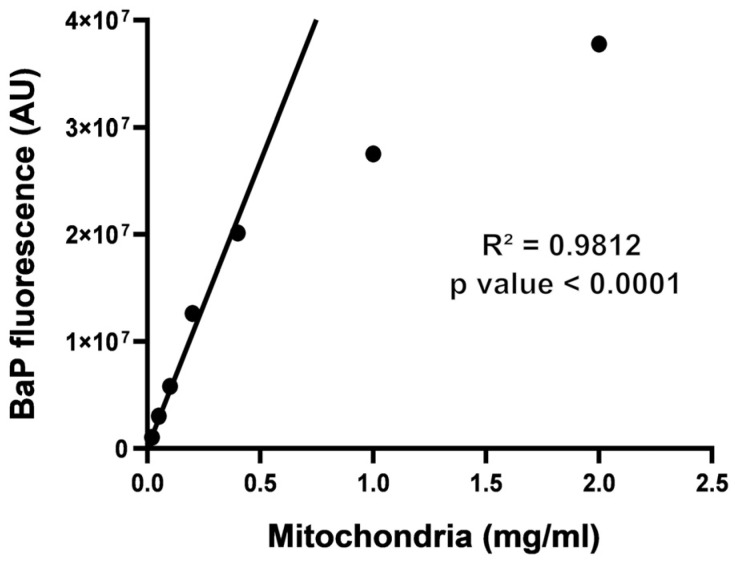
Affinity of BaP for mitochondria. The affinity of BaP for mitochondria was assessed by incubating different concentrations of mitochondria with BaP. The unbound BaP was discarded after 2 h of incubation, and the fluorescence of mitochondria was analysed (Em: 377/Ex: 418 nm). First, the basal fluorescence of mitochondria was subtracted from the data. Since BaP emits fluorescence in blue wavelengths, this method allows to see the BaP bound to mitochondria. Quantification of the fluorescence signal shows that some amount of BaP binds to mitochondria in a dose-dependent manner. The linear regression shows a positive correlation of BaP affinity up to 0.4 mg/mL mitochondria (R^2^ = 0.9812; *p*-value < 0.0001), demonstrating that BaP has an affinity for mitochondria (*n* = 3).

Isolated mitochondria were diluted at different concentrations and incubated with excess molar BaP (5 µM). We used the ability to detect BaP fluorescence (Ex: 377 nm, Em: 418 nm) to measure the capacity of BaP to bind mitochondria. The amount of BaP-related fluorescence increases with mitochondrial concentration and the linear positive correlation up to 0.4 mg/mL of mitochondria (R^2^ = 0.9812; *p*-value < 0.001), where it seems to saturate. Figure 1 indicates that BaP is indeed binding to mitochondria. This is consistent with the lipophilic properties of BaP, which cause it to bind to the mitochondrial membranes.

### 2.2. Lipid Peroxidation Is Induced in Isolated Mitochondria and Cardiolipin-Containing Multilamellar Vesicles (MLVs) Exposed to BaP/UVAssl

**Figure 2:**
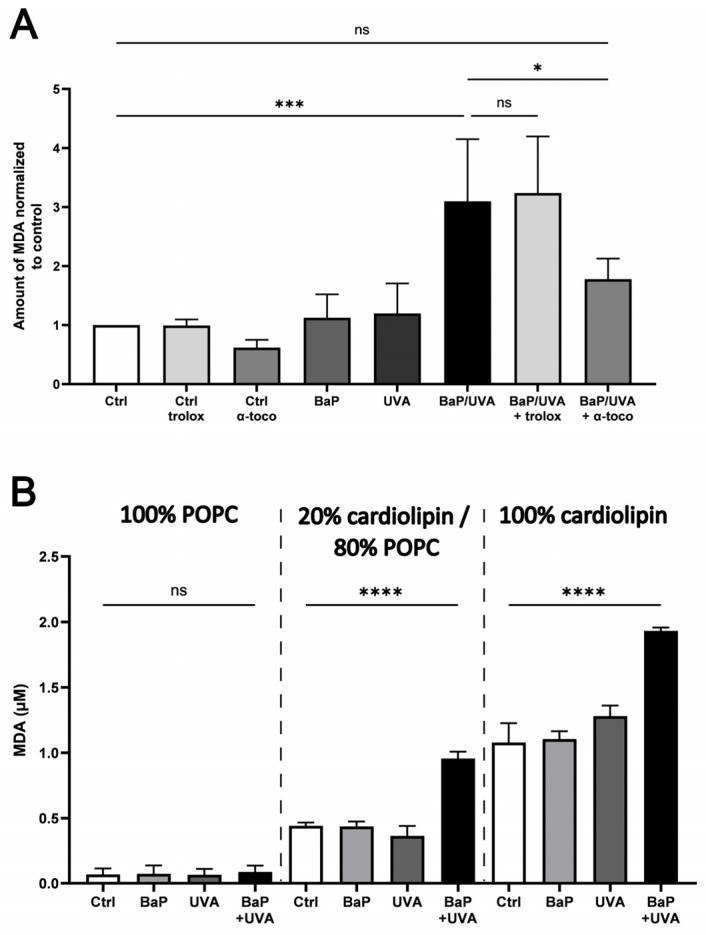
Lipid peroxidation in BaP- and/or UVAssl-exposed isolated mitochondria and cardiolipin-containing lipid vesicles. Lipid peroxidation was assessed by measuring MDA, a by-product of lipid peroxidation. (**A**) Isolated mitochondria pre-treated or not with antioxidants were incubated with 500 nM of BaP and/or 200 kJ/m^2^ of UVAssl. Exposure to BaP or UVAssl alone does not lead to the significant generation of MDA. BaP and UVAssl co-exposure leads to a significant production of MDA (3-fold the control level). α-Tocopherol significantly decreases BaP/UVAssl-induced MDA production, whereas Trolox has no effect. (**B**) Lipid peroxidation was determined in MLVs containing cardiolipin. MLVs containing 100% POPC, 100% cardiolipin or a mix of cardiolipin and POPC at a ratio of 20:80 were exposed to 500 nM BaP and/or to 200 kJ/m^2^ UVAssl. No MDA formation could be measured in MLVs containing 100% POPC exposed to BaP and/or UVAssl. For MLVs containing a mix of cardiolipin and POPC at a ratio of 20:80, the treatment with either BaP or UVAssl does not affect the basal MDA levels. However, when co-exposed to BaP and UVAssl, we found a significant increase (2-fold) in lipid peroxidation. MLVs containing 100% cardiolipin have higher basal levels of MDA, with a significant increase only when they are co-exposed to BaP and UVAssl. Values are mean ± SD (*n* = 3). ns = non-significant; * *p* < 0.05; *** *p* < 0.001; **** *p* < 0.0001.

Isolated mitochondria were treated with BaP (500 nM) and/or UVAssl (200 kJ/m^2^) (Figure 2A). Lipid peroxidation was assessed by measuring malondialdehyde (MDA), a by-product of lipid peroxidation. BaP or UVAssl alone do not significantly affect the basal level of MDA and, consequently, lipid peroxidation. Co-exposure to BaP and UVAssl leads to a significant increase in lipid peroxidation in mitochondria. Trolox, a hydrophilic antioxidant, does not prevent lipid peroxidation induced by co-exposure to BaP/UVAssl, but its lipophilic counterpart, α-tocopherol, decreases it 2-fold. 

Multilamellar vesicles (MLVs) containing 1-palmitoyl-2-oleoyl-glycero-3-phosphocholine (16:0–18:1 PC) (POPC) and/or cardiolipin were treated with BaP (500 nM) and/or UVAssl (200 kJ/m^2^) (Figure 2B). MLVs containing 20% cardiolipin/ 80% POPC (20/80 mix) represent the ratios found in the inner mitochondrial membrane. The MLVs containing 100% POPC or 100% cardiolipin were used as controls. No significant amount of MDA was measured in the MLVs containing 100% POPC treated with BaP and/or UVAssl (Figure 2A). The MLV 20/80 mix does not generate MDA when treated with BaP or UVAssl alone. However, when co-exposed to BaP and UVAssl, the 20/80 mix and 100% cardiolipin generate a significant amount of MDA (around 2-fold more than the control). MLVs constituted of cardiolipin alone have a higher basal (untreated) MDA level than the other vesicles, most likely due to their high potential sensitivity to oxidation because of their higher number of unsaturated bonds. 

### 2.3. Co-Exposure to BaP/UVAssl Destabilizes Mitochondrial Inner Membranes and Leads to Mitochondrial Fission

**Figure 3:**
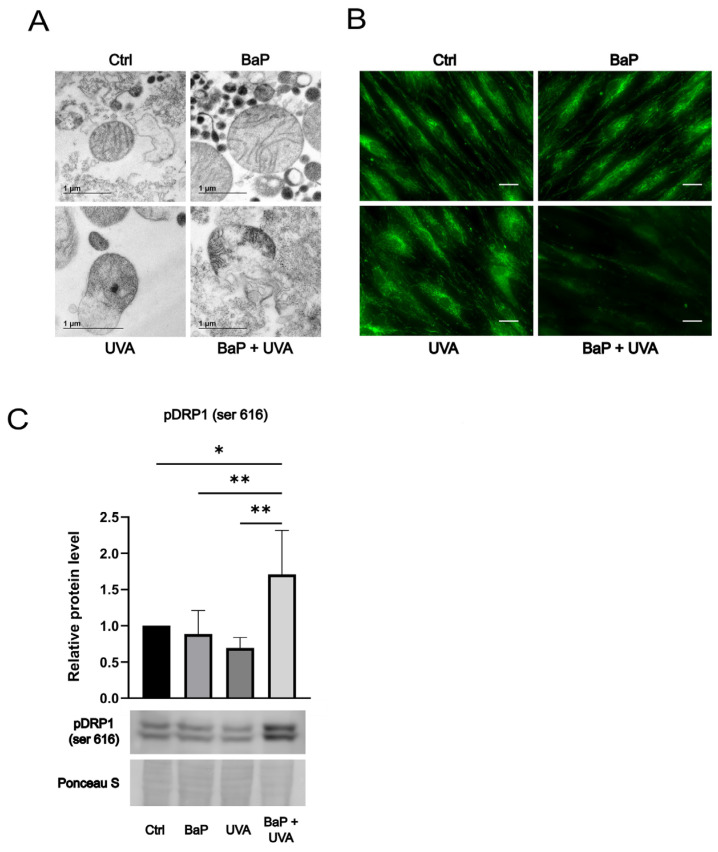
Impact of BaP/UVAssl on mitochondrial morphology and network. (**A**) The morphology of mitochondria treated with 500 nM of BaP and/or exposed to 200 kJ/m^2^ of UVAssl was visualized using transmission electronic microscopy (TEM). The morphology of mitochondria treated with BaP alone is not visually different from the untreated mitochondria. We can observe membrane distortion in mitochondria treated with UVAssl alone, but the membrane still seems intact. Co-exposure to BaP and UVAssl leads to a clear loss of mitochondrial membrane integrity. (**B**) The qualitative assessment of mitochondrial fission was determined using a mitochondrial fluorescent probe (MitoTracker Green). We can visually observe that co-exposure to BaP (100 nM) and UVAssl (20 kJ/m^2^) leads to a loss of the Mitotracker signal, suggesting fission of the mitochondrial network. This effect is not observed when cells are treated with only BaP or UVAssl. Scale bar = 20 µm. (**C**) Protein analysis by Western blot of phosphorylated (ser616) DRP1 (pDRP1; fission) of a protein extract from BaP- (100 nM) and/or UVAssl-exposed (20 kJ/m^2^) skin fibroblasts. The quantification of the Western blot shows an increase in fission, as evidenced by the increase in pDRP1 in BaP/UVAssl-exposed cells but not in the other conditions (untreated, BaP alone and UVAssl alone). All values are mean ± SD (*n* = 5). * *p* < 0.05; ** *p* < 0.005.

Isolated mitochondria were co-exposed to BaP (500 nM) and UVAssl (200 kJ/m^2^), and mitochondrial morphology was qualitatively assessed by transmission electronic microscopy (Figure 3A). BaP treatment alone does not visually affect mitochondrial morphology. UVAssl treatment creates some outer membrane distortions, but mitochondria keep their global morphology. When mitochondria are co-exposed to BaP and UVAssl, we can observe a clear loss of membrane integrity, with an important distortion of the cristae. 

The Mitotracker fluorescence intensity decreases in BaP/UVAssl co-exposed fibroblasts (Figure 3B), which indicates an alteration of the mitochondrial network by photo-pollution. Protein levels of pDrp1 (ser616), a marker of mitochondrial fission (Figure 3C), and MFN1, a marker of mitochondrial fusion (Figure S2), were determined in fibroblasts treated with BaP and/or UVAssl. Mitochondrial fission is mediated by Drp1, which is phosphorylated on serine 616 (pDRP1) when recruited to mitochondria [38]. MFN1 is a mitochondrial transmembrane protein essential for the fusion of mitochondria [38]. BaP and UVAssl alone do not significantly affect the levels of pDRPHowever, co-exposure to BaP and UVAssl leads to a significant increase in pDRP1 (mitochondrial fission). 

### 2.4. Mitochondrial Membrane Potential and Metabolism Is Depleted by BaP/UVAssl

**Figure 4:**
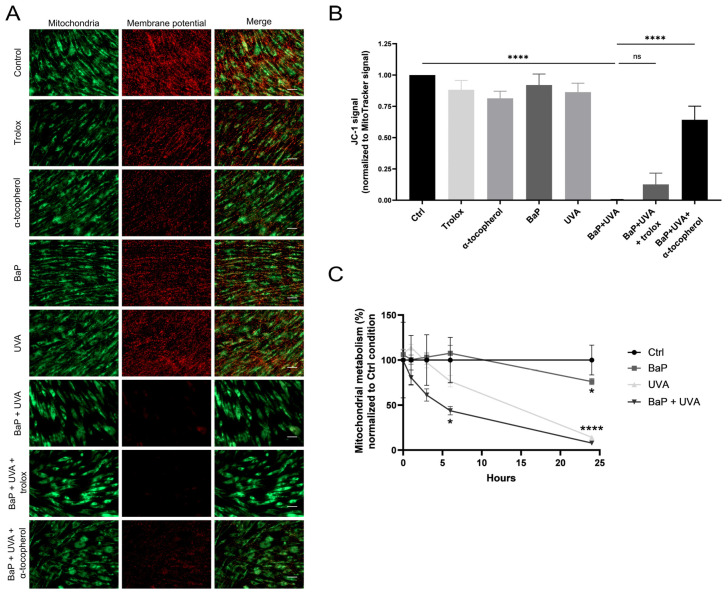
Alteration of mitochondrial ΔΨ and metabolism following exposure to BaP and UVAssl. (**A**) Mitochondrial ΔΨ was measured using the fluorescent probe JC-1. Fibroblasts, pre-treated or not with antioxidants, were exposed to 500 nM of BaP and/or to 25 kJ/m^2^ of UVAssl and then stained with JC-1 (membrane potential; red) and Mitotracker (mitochondria; green). Scale bar = 50 µm. (**B**) Quantification of the fluorescent signal indicates that co-exposure to BaP/UVAssl leads to a significant loss of ΔΨ, whereas BaP or UVAssl alone do not significantly influence basal ΔΨ. The use of α-tocopherol, but not Trolox, prevents ΔΨ loss. All values are mean ± SD (*n* = 4). (**C**) Mitochondrial metabolism was assessed using the MTS assay on enriched mitochondrial fractions exposed to 500 nM of BaP and/or to 20 kJ/m^2^ of UVAssl. Exposure to BaP alone has no effect on mitochondrial metabolism, and the UVAssl irradiation leads to a non-significant decrease in mitochondrial metabolism. Co-exposure to BaP/UVA leads to a significant decrease in mitochondrial metabolism at 6 h post-exposure. All values are mean ± SD (*n* = 3). ns = non-significant; * *p* < 0.05; **** *p* < 0.0001.

Mitochondrial membrane potential (ΔΨ) was measured in fibroblasts exposed to BaP (500 nM) and/or UVAssl (25 kJ/m^2^) (Figure 4A,B). UVAssl or BaP treatment alone did not significantly affect ΔΨ. However, when combined, UVAssl and BaP lead to a 3-fold decrease in ΔΨ. The potential of Trolox and α-tocopherol to prevent this effect was tested. Trolox had no significant effect, but α-tocopherol led to the significant restoration of ΔΨ in BaP/UVAssl-treated fibroblasts. 

The consequence of exposure to BaP (500 nM) and/or UVAssl (20 kJ/m^2^) on mitochondrial metabolism was determined using an MTS assay on isolated mitochondria (Figure 4C). The MTS assay measures the conversion of MTS tetrazolium to formazan dye by mitochondrial reductase and is thus a direct indicator of mitochondrial metabolism. Metabolism was monitored 0, 1, 3, 6 and 24 h post-exposure (Figure 4C). BaP alone did not influence mitochondrial metabolism. UVAssl exposure leads to a slight but non-significant decrease in metabolism at 6 h and to the complete and significant loss of metabolism 24 h post-exposure. When mitochondria are co-exposed to BaP/UVAssl, it generates a decrease in metabolism, which is significant at 6 h. 

## 3. Discussion

The implication of mitochondria in premature skin aging is well described, and several studies have investigated the impact of UVAssl [5,6,9] and pollutants [39,40,41] on this organelle. In this project, we aimed to determine the effect of co-exposure to pollution and UV rays on mitochondria, i.e., mitochondrial membranes and mitochondrial functions. As the study model, skin fibroblasts as well as enriched mitochondrial fractions were exposed to BaP and/or UVAssl. BaP was used because of its recognized toxicity [23] but also because it has been shown to absorb UVA wavelengths [22,29,30,42]. 

Berneburg et al. [7] have shown that chronic UV exposure is implicated in premature skin aging via ROS production and the accumulation of mitochondrial DNA deletions. We and others have previously highlighted that concomitant exposure to BaP and UVAssl leads to a synergistic toxicity, which generates important levels of ROS in skin cells [22,33,43,44]. It became interesting to determine whether the BaP/UVAssl synergy is specifically affecting mitochondrial integrity through ROS induction, which would bring further evidence of a link between photo-pollution and premature aging. We have previously shown that the BaP/UVAssl synergy creates an important amount of ROS, which leads to lipid peroxidation [43]. In this paper, we aimed to determine whether this important amount of ROS and lipid peroxidation was targeted toward mitochondrial membranes.

We show that the lipophilic BaP has an affinity for mitochondria (Figure 1), which is consistent with the fact that this organelle has two lipid membranes. We found an important induction of lipid peroxidation in isolated mitochondria co-exposed to UVAssl and BaP (Figure 2A). This mitochondrial oxidation can be prevented using α-tocopherol, a lipophilic antioxidant, but not Trolox, its hydrophilic counterpart. This confirms the lipid localization of the oxidation, but it also opens potential for a photoprotection strategy. Indeed, α-tocopherol could be used to prevent mitochondrial damage induced by photo-pollution exposure. 

The inner mitochondrial membrane contains up to 18% of cardiolipin, a specific mitochondrial lipid which contains four poly-unsaturated acyl chains [45]. Cardiolipins are important for the formation of mitochondrial cristae due to their conical structure. They allow the layover of the inner mitochondrial membrane, help stabilize respiratory protein super-complexes and serve as anchorage for mtDNA during replication [46,47]. Moreover, there is evidence that cardiolipins are implicated in the initiation of cytochrome c release and in the creation of permeability transition pores for apoptotic factors [45,46,48]. Poly-unsaturated acyl chains of cardiolipins are highly prone to lipid peroxidation. Our results indeed confirm that cardiolipins are a target of lipid peroxidation induced by photo-pollution (Figure 2B).

To determine lipid peroxidation induction following UVAssl and BaP exposure, we used MDA, a by-product of lipid peroxidation (Figure 2). In addition of being a marker of lipid peroxidation, MDA is an aldehyde that can react with guanine to generate M1dG, a DNA bulky adduct, which can ultimately lead to frameshift mutations and base pair substitutions [49,50,51]. M1dG can be repaired by nucleotide excision repair, but since this repair system is absent in mitochondria [52], it can theoretically accumulate in mtDNA. The alteration to mtDNA has been associated with photo-aging [5,6,9], and it can thus be speculated that photo-pollution could contribute to premature aging through the alteration of mtDNA.

To qualitatively assess the toxic synergy of BaP and UVAssl on mitochondrial membranes, we observed isolated mitochondria exposed to both agents using transmission electronic microscopy (Figure 3A). Our results clearly show an important destabilization of mitochondrial membranes with visual loss of inner and outer membrane integrity when exposed to BaP and UVAssl. This important alteration is observed on isolated mitochondria, but the cellular reality is that mitochondria are living in a well-organized network. Here, we show that the stress generated by UVAssl/BAP co-exposure leads to fission of the mitochondrial network (Figure 3B,C). This is further confirmed by a decreased mitochondrial fusion (Figure S2). The mitochondrial fission confirms the toxic BaP/UVAssl synergy at the mitochondrial level. A recent study has shown that autophagy was downregulated following exposure to UV rays and BaP [32]. Thus, we can predict that cells exposed to UV/BaP became less efficient in recycling damaged mitochondria, which adds to the implication of mitochondrial damage.

Lipid peroxidation in the inner mitochondrial membrane and the alteration of the mitochondrial network by co-exposure to UVAssl/BaP could impact mitochondrial activity. We indeed showed that co-exposure to BaP and UVAssl caused a loss of mitochondrial membrane potential (Figure 4A,B) associated with a decrease in mitochondrial metabolism (Figure 4C). Moreover, we showed that α-tocopherol significantly prevents membrane potential loss caused by UVAssl/BaP co-exposure, which highlights the involvement of UVAssl/BAP-induced lipid peroxidation in the loss of membrane potential. 

Other teams have previously shown mitochondrial fission, the alteration of mitochondrial respiration and the loss of mitochondrial membrane potential in cells exposed to UV rays or pollution [12,13,22,24,39,41]. However, to our knowledge, we are the first to demonstrate the effect of BaP/UVAssl synergy on mitochondria and the implication of lipid peroxidation in this toxicity. We observed a loss of mitochondrial membrane potential leading to a decrease in mitochondrial metabolism when mitochondria are co-exposed to BaP and UVAssl, highlighting the synergy between both environmental factors. The synergistic effect is, at least in part, caused by lipid peroxidation, which takes place in cardiolipin-rich inner mitochondrial membranes. Since mitochondria are strongly implicated in skin aging, the preservation of mitochondrial integrity and functions through prevention strategies targeting mitochondria is crucial.

## 4. Material and Methods

All experiments performed in this study were conducted in accordance with our institution’s guidelines and the Declaration of Helsinki.

### 4.1. Cell Culture

Five different strains of human diploid dermal fibroblasts from skin biopsies of healthy women (mastectomy) were used (age 18–25 years). Immortalized human embryonic kidney cells (HEK-293T) (American Type Culture Collection #CRL-3216) were used for the extraction of mitochondria. Cells were cultured in Dulbecco’s modified Eagle’s Medium (DMEM; Wisent Inc., Saint-Jean-Baptiste, QC, Canada) supplemented with 5% foetal bovine serum (FBS) (Sigma-Aldrich, Oakville, ON, Canada) and 1% penicillin/streptomycin (Wisent Inc., Saint-Jean-Baptiste, QC, Canada) at 37 °C, 5% CO_2_. 

### 4.2. Mitochondrial Extraction

An enriched mitochondrial fraction was obtained as previously described [53]. Briefly, confluent HEK-293T cells were harvested and resuspended in a mitochondria isolation buffer (Mi). Cells were then crushed using a Teflon pestle and then filtered using 40 µm, 10 µm and 5 µm polyethylene terephthalate (PET) filters (pluriSelect, El Cajon, CA, USA). After centrifugation, mitochondria were resuspended in the Mi and diluted at a concentration of 2 mg/mL.

### 4.3. BaP Affinity to Mitochondria

The enriched mitochondrial fraction was diluted in the Mi between 2 and 0.02 mg/mL. Each fraction was exposed to 5 µM BaP. Samples were then incubated for 2 h at 37 °C, 5% CO_2_. Mitochondrial fractions were then centrifuged at 8000 G; the supernatant containing the unbound BaP was discarded and the pellet was resuspended in the Mi. The fluorescence of the mitochondria containing or not containing BaP was determined at Em: 377/Ex: 418 nm using a CytoFluor^®^ Series 4000 multi-well fluorescence plate reader (Applied Biosystems, Woburn, MA, USA). 

### 4.4. Pollutants and UVA Exposure

Confluent monolayers of fibroblasts or isolated mitochondria were pre-treated with BaP (0-500 nM) diluted in phosphate buffered saline (PBS) or Mi for 30 min in the dark and then exposed to UV light. Prior to this step, BaP (Sigma–Aldrich, Oakville, ON, Canada) was dissolved in dimethyl sulfoxide (DMSO).

For irradiation, an Oriel solar simulator (ssl) 1.6 kW with an ozone-free xenon short arc lamp equipped with a 1.5 G filter was used (Newport, Irvine, CA, USA). Wavelengths under 340 nm (UVB and UVA2) were blocked using a CGA-345 filter (Schott, Lebanon, PA, USA) (Figure S1). UVA irradiance (approximately 2 mW/cm^2^ at cell surface) was measured prior to each experiment using a UVP UVX Radiometer (Thermo-Fisher, Mississauga, ON, Canada). After irradiation, PBS or Mi +/− pollutants were discarded. Fresh DMEM or Mi was added to cells or mitochondria, respectively, and incubated at 37 °C, 5% CO_2_ for the indicated time. 

### 4.5. Antioxidant Treatments

Cells were treated with 100 µM α-tocopherol (vitamin E; Sigma-Aldrich, ON, Canada) (α-toco) or 100 µM Trolox (Abcam, Cambridge, UK) 16 h before the pollutant treatment. Antioxidants were also added at the same concentration as PBS during the light exposure and then to the medium after irradiation. For mitochondria, antioxidants were added to the Mi 30 min before irradiation. After irradiation, mitochondria were centrifuged and resuspended in fresh Mi +/− antioxidants.

### 4.6. MDA Evaluation

Isolated mitochondria, pre-treated or not with antioxidants, were incubated with BaP (500 nM) in the Mi for 30 min and then exposed to 200 kJ/m^2^ UVAssl. After irradiation, the Mi was discarded and replaced with Mi with or without antioxidants. After a 2-h incubation at 37 °C, 5% CO_2_, MDA formation was assessed using the lipid peroxidation (MDA) assay kit (Abcam, Cambridge, UK; Ab118970) with some modifications. Briefly, the modifications are the following: after cell lysis, thiobarbituric acid (TBA) was added to the samples for 1 h at 95 °C. MDA production was detectable by fluorescence using a CytoFluor^®^ Series 4000 multi-well fluorescence plate reader (Applied Biosystems, Waltham, MA, USA) (Ex: 532 nm/Em: 572 nm). The results were normalized to the control condition for each replicate.

### 4.7. Preparation of MLVs

POPC (Avanti Polar Lipids, Alabaster, AL, USA) and cardiolipin (Avanti Polar Lipids, Alabaster, AL, USA) were mixed at different molar ratios (cardiolipin:POPC 100:0, 0:100 or 20:80) in chloroform. The chloroform was removed using a stream of argon, and lipid films were then lyophilized under vacuum for 1 h. Dried lipids were resuspended in water and vortexed to obtain MLVs. For the MDA assessment of lipid vesicles, TBA was directly added to the different MLV mix and then incubated at 95 °C for 1 h.

### 4.8. Transmission Electron Microscopy (TEM)

Isolated mitochondria were incubated with BaP (500 nM) in the Mi for 30 min and then exposed to 200 kJ/m^2^ UVAssl. After irradiation, mitochondria were centrifuged at 8000 G for 10 min, resuspended in PBS and incubated for 2 h at 37 °C, 5% CO_2_. Mitochondria were then centrifuged and resuspended in a fixation solution (containing 2.5% glutaraldehyde and 0.1 M cacodylate) for 24 h. Mitochondria were then centrifuged and resuspended in 0.1 M cacodylate. Mitochondria were then examined using a transmission electronic microscope, JEOL 1230 (CHM-0166B, JEOL, Tokyo, Japan). 

### 4.9. Mitochondrial Fission

Qualitative assessment by microscopy: Three different strains of fibroblasts were treated with BaP (100 nM) and irradiated to UVAssl (20 kJ/m^2^). Two hours post-treatment, cells were stained with Mitotracker Green (160 nM; Invitrogen, Waltham, MA, USA) diluted in PBS for 30 min at 37 °C to qualitatively assess the mitochondrial network by microscopy.

Quantitative assessment of mitochondrial network by Western blot: Five strains of fibroblasts were treated with 100 nM BaP and 20 kJ/m^2^ of UVAssl. Cells were harvested and resuspended in RIPA buffer (1% NP40, 0.5% sodium deoxycholate, 0.1% SDS in PBS, pH 7.4) containing a protease inhibitor cocktail (Roche Applied Science, Mississauga, ON, Canada). Twenty micrograms of total proteins for each sample were used for the blot. A primary antibody against pDRP1 (ser616) (#3455S; Cell Signaling Technology, Danvers, MA, USA) 1:1000 was used for an overnight incubation at 4 °C, and secondary anti-rabbit HRP-conjugated IgG antibody (Sigma-Aldrich, Oakville, ON, Canada; diluted at 1:5000) was incubated for 1 h at room temperature. Proteins were visualized using chemiluminescence reagents (Thermo Fisher Scientific, Mississauga, ON, Canada) with a C-DiGit Blot Scanner (LI-COR Biosciences, Lincoln, NE, USA) and analysed by Image Studio Lite software version 5.0 (LI-COR Biosciences; https://www.licor.com/bio/image-studio-lite/, accessed on 2 August 2024).

### 4.10. Membrane Potential Assessment

Four different strains of fibroblasts were treated with BaP (500 nM) and irradiated to UVAssl (25 kJ/m^2^). Two hours post-treatment, cells were stained with JC-1 dye (2.5 µM; Invitrogen, Waltham, MA, USA) and Mitotracker DeepRed (80 nM; Invitrogen, Waltham, MA, USA) diluted in PBS for 30 min at 37 °C to assess the potential of the inner mitochondrial membrane. Cells were then analysed by microscopy and analysed to generate the ratio between JC-1 and MitoTracker.

### 4.11. Metabolism Assessment

The metabolism of isolated mitochondria treated with BaP (500 nM) and irradiated to UVAssl (20 kJ/m^2^) was assessed using an MTS cell proliferation assay (CellTiter 96^®^ AQueous Non-Radioactive Cell Proliferation Assay; Promega, Fitchburg, WI, USA) with some modifications. Briefly, the experiment was performed according to the manufacturer’s protocol at 0, 1, 3, 6 and 24 h post-exposure. Before the addition of the MTS solution to the samples, 1 mM malate, 1 mM pyruvate and 2.5 µM ADP were added to allow the mitochondria to realize metabolism. A colorimetric analysis was realized using a microplate reader (BioRad 550 Microplate Reader; BioRad, Hercules, CA, USA). Values were corrected with background absorbance and the average of the control was used as a baseline.

### 4.12. Statistical Analysis

Statistical analyses were performed using GraphPad Prism version 9 software (GraphPad Software, San Diego, CA, USA). Differences between conditions were assessed using a one-way analysis of variance (ANOVA1) with Tukey HSD procedure as a post hoc test. A *p*-value ≤ 0.05 was defined as statistically significant.

## Data Availability

The data presented in this study are available on request from the corresponding author.

## Supplementary Materials

The following supporting information can be downloaded at: https://www.mdpi.com/article/10.3390/ijms25179146/s1.
