# Supplementary material for: Synergistic Toxicity of Pollutant and Ultraviolet Exposure from a Mitochondrial Perspective
Source: Int J Mol Sci. 2024 Aug 23;25(17):9146. doi: 10.3390/ijms25179146 (PMC11394743; doi:10.3390/ijms25179146)
Supplement: Supplementary file 1 [file ijms-25-09146-s001.zip › ijms-3184170-supplementary.pdf]

Supplementary material

Synergic toxicity of pollutants and ultraviolet from a mitochondrial perspective

Eloïse Larnac[1,2], Sébastien Méthot[1,2], Frédéric Pelchat[1], Marc-Antoine Millette[1], Alicia Montoni[,2,3], Christian Salesse[1,3], Valérie Haydont[4], Laurent Marrot[4], Patrick J. Rochette[1,2,3,*]

1. Centre de Recherche du CHU de Québec – Université Laval, Axe Médecine Régénératrice, Hôpital du Saint-Sacrement, Québec, Canada
2. Centre de recherche en organogénèse expérimentale de l'Université Laval/LOEX, Québec, Canada
3. Université Laval, Faculté de Médecine, Département d'Ophtalmologie, Université Laval, Québec, Canada.
4. Advanced Research, L'OREAL Research & Innovation

* Corresponding author:
Pr. Patrick J. Rochette
Centre de recherche du CHU de Québec – Université Laval
Axe médecine Régénératrice
Hôpital du Saint-Sacrement, Bureau H2-10
1050 Chemin Sainte-Foy, Québec, Qc, Canada, G1S 4L8
Phone: (418) 682-7568
E-mail: Patrick-J.Rochette@crchudequebec.ulaval.ca

MATERIAL AND METHODS

Mitochondrial fusion

Quantitative assessment of mitochondrial fusion was performed by western blot. Primary antibody against MFN1 (D6E2S) (#14739; Cell Signaling Technology, Danvers MA, USA) 1:1000 was used for an overnight incubation at 4 °C and secondary anti-rabbit HRP-conjugated IgG antibodies (Sigma-Aldrich, Oakville, ON, Canada; diluted at 1:5000) was incubated 1 h at room temperature. Proteins were visualized using chemiluminescence reagents (Thermo Fisher Scientific) with a C-DiGit Blot Scanner (LI-COR Biosciences, Lincoln, NE, USA) and analysed by Image Studio Lite software version 5.0 (LI-COR Biosciences).

RESULTS

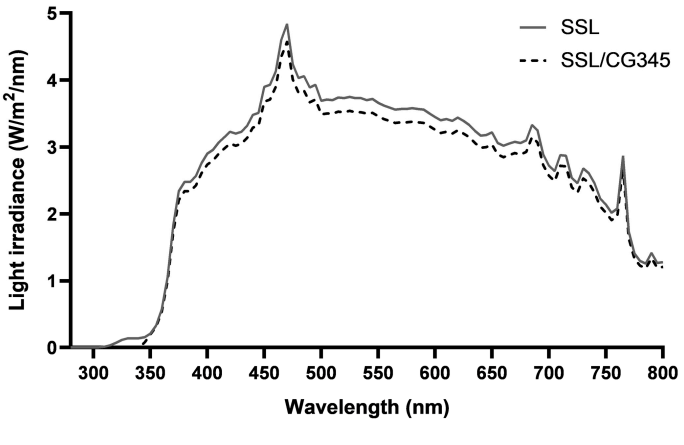

Figure S1: Emission spectra of the SSL with filter CGA345. Light source output consists of an Oriel solar simulator (SSL) with an ozone-free xenon short arc 1.6 kW lamp combined with an air

mass 1.5 G (AM1.5 G) filter (grey solid line, ssl) and a Schott CGA-345 (black dashed line, UVAssl)

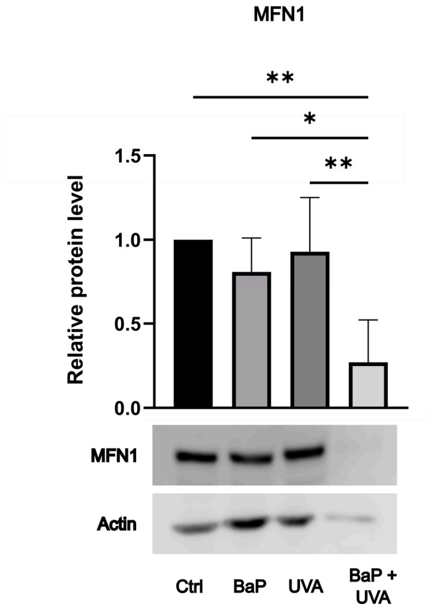

Figure S2. Mitochondrial fusion when exposed to BaP and/or UVAssl. Western blot protein analysis of MFN1 of skin fibroblasts exposed to BaP and/or UVAssl. Quantification of the blot shows a reduction of mitochondrial fusion in BaP (200 nM)/UVAssl (25 kJ/m^2) exposed cells but not in the other conditions (untreated, BaP alone and UVAssl alone). All values are mean ± SD (N=4). *p<0.05; **p<0.005.
